# Multifocal Rosai Dorfman disease and simultaneous endometrioid ovarian cancer: a case report

**DOI:** 10.1186/s13256-025-05772-4

**Published:** 2026-03-31

**Authors:** Nele Graf, Teresa Halbsguth, Sven Becker, Morva Tahmasbi Rad

**Affiliations:** 1 Department of Obstetrics and Gynecology, University Hospital Frankfurt, Goethe University, 60598 Frankfurt, Germany; 2 Department of Medicine, Hematology/Oncology, University Hospital Frankfurt, Goethe University, 60598 Frankfurt, Germany

**Keywords:** Rosai Dorfman disease, Cobimetinib, Ovarian cancer, KRAS mutation

## Abstract

**Background:**

Rosai Dorfman disease is a rare histiocytosis, which is characterized by accumulation of histiocytes in lymph nodes, but also in extranodal locations. Histologically, one of the key features of Rosai Dorfman disease is emperipolesis. Treatment options involve surgical resection, radiation, chemotherapy, corticosteroids, and immunotherapy. Rosai Dorfman disease treatment depends on disease location and symptomatology. Patients with mild nodal or cutaneous involvement usually have a self-limited course and require observation only. The exact pathogenesis of Rosai Dorfman disease is still being evaluated, but mutations of the Kirsten rat sarcoma virus gene are found in up to a third of the patients and are linked to multifocal disease. Recent publications and case reports have shown that treatment with the mitogen-activated protein kinase kinase inhibitor Cobimetinib is a possible option in Rosai Dorfman disease patients with Kirsten rat sarcoma virus gene mutation.

**Case presentation:**

In this case report, we are presenting and discussing the case of a 47-year-old Caucasian female patient with multifocal Rosai Dorfman disease and simultaneous endometrioid ovarian cancer, who underwent median laparotomy for tumor debulking of the endometrioid ovarian cancer and in which a Kirsten rat sarcoma virus gene mutation was detected and a treatment with Cobimetinib was initiated, leading to sustained remission.

**Conclusion:**

While there are case reports on patients with Rosai Dorfman disease with a simultaneous cancer diagnosis, and while some authors describe Rosai Dorfman disease as a possible neoplastic process itself, it remains unclear if there is a causal association between Rosai Dorfman disease and cancer.

**Supplementary Information:**

The online version contains supplementary material available at 10.1186/s13256-025-05772-4.

## Background

Rosai Dorfman disease (RDD, also known as Rosai Dorfman Syndrome or Rosai Dorfman Destombes disease) is a rare Non-Langerhans-cell histiocytosis, which is characterized by lymphadenopathy and a wide spectrum of clinical manifestations through accumulation of histiocytes in various places. Approximately 43% of the patients have extranodal manifestations [1]. RDD was first described by Rosai and Dorfman in 1969 [2]. RDD is part of the myeloproliferative disorders. Even though it does have features of transformed cells, it is not described as cancer because of a low proliferative index of the histiocytes [3, 4]. With an incidence of 1:200,000 cases it is a rare disease [5]. Patients with RDD are typically young African American children or young adults, who often present with massive cervical lymphadenopathy, but extranodal and cutaneous lesions are also common [5, 6]. Histopathologically, RDD is usually characterized by large nucleolated and hypochromatic histiocytes, positivity for S100 protein and emperipolesis [7]. Emperipolesis is the presence of a viable cell within the cytoplasm of another cell [8]. Treatment of RDD involves complete surgical resection in patients with unifocal disease, corticosteroids, sirolimus, radiation, immunotherapy, or chemotherapy including anthracyclins and alkaloids in patients with extensive disease. Self-limitation is also possible in patients with mild nodal and/or cutaneous involvement [6, 9, 10]. Targeted therapies such as the mitogen activated protein kinase kinase (MEK) inhibitor Cobimetinib are also used in patients with mutation in the Kirsten rat sarcoma virus (KRAS) gene [11]. RDD can be associated with autoimmune diseases including lupus erythematodes, autoimmune hemolytic anemia, or juvenile idiopathic arthritis as well as different types of neoplasia [1, 6, 12]. In the following case report, we want to present and discuss the case of a 47-year-old female patient with extensive multifocal RDD of several locations and simultaneous endometrioid ovarian cancer.

## Case presentation

A middle-aged Caucasian female patient firstly presented to the ear nose and throat (ENT) department in 2017 with restricted nasal breathing. The patient’s medical history was notable only for a hemithyroidectomy performed during adolescence in Bulgaria due to goiter; there were no other known comorbidities and the family history was unremarkable. She was diagnosed with nasal polyposis and a pansinus surgery was performed. Histopathological examination revealed a lymphocyte- and plasma cell-rich lesion with emperipolesis, consistent with RDD. The patient was subsequently referred to our hemato-oncological department for further evaluation and management. Given the absence of symptoms and lymphadenopathy, a watch-and-wait strategy was adopted.

In early 2022, she was referred to our endocrinology department for evaluation of a previously diagnosed latent hyperthyroidism treated with 14 milligrams (mg) of carbimazole. In the interim, she had undergone multiple procedures at other hospitals, including repeat pansinus surgery with partial mastoidectomy and conchotomy, cervical lymph node excision, partial cheek resection, and maxillary sinus fenestration due to left-sided neck swelling. She had also undergone an orbital biopsy via anterior orbitotomy. According to the reports, histology again showed plasma cell- and histiocyte-rich infiltrates, though RDD was not explicitly mentioned. A subsequent skeletal scintigraphy performed externally demonstrated tracer uptake in the facial bones and distal femoral shafts. Thyroid scintigraphy—initiated because of latent hyperthyroidism—revealed hypofunctional areas without features of autoimmune thyroiditis.

**Fig. 1:**
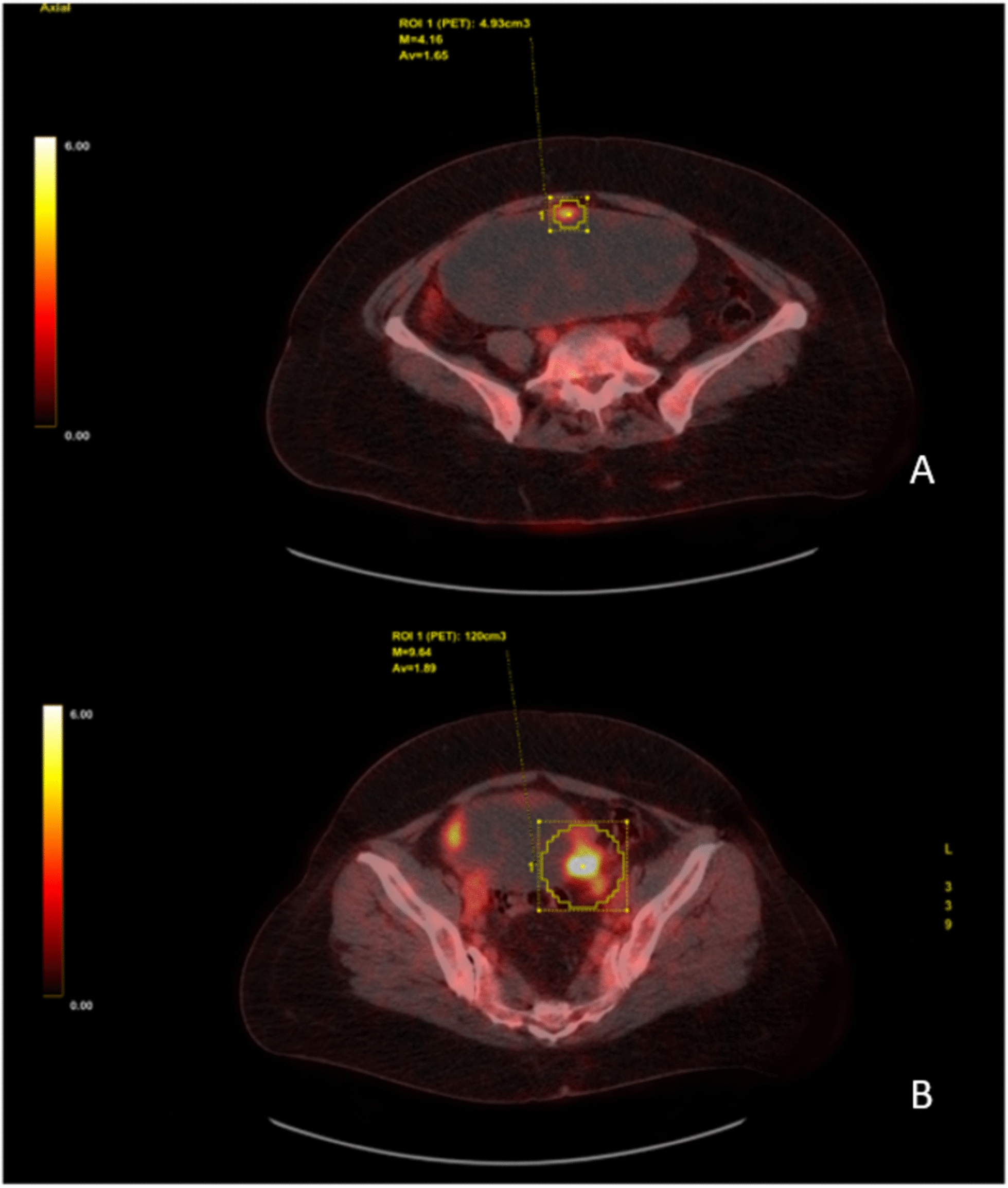
Positron emission tomography–computed tomography scan. **A** Tumor mass with cystic-liquid parts in lower abdomen presenting with elevated glucose utilization at the margins. Pathological results revealed an endometriod borderline tumor with transition into a moderate degree endometriod ovarian cancer. **B** Hypermetabolic mass para-iliacal on the left side. Pathological results revealed RDD

Upon presentation to our endocrinology department, laboratory testing showed euthyroid function, and carbimazole was discontinued. Repeat thyroid scintigraphy demonstrated mild disseminated autonomy without typical signs of Graves’ disease or focal autonomy. Thyroid biopsy confirmed RDD. On physical examination, no palpable lymphadenopathy was detected; however, inspection revealed swelling of the eyelid and thyroid region. A positron emission tomography–computed tomography (PET–CT) scan showed hypermetabolic RDD manifestations in the following locations: intraorbital, right maxillary sinus, inferior nasal concha, right neck, thyroid extending into the mediastinum, and the medullary space of the lower extremities. In addition, a hypermetabolic left parauterine mass (16.7 × 7.8 cm) with cystic-solid morphology (Fig. 1), increased peripheral glucose uptake, and radiological features suspicious for malignancy and a hypermetabolic leftsided parailiac mass was detected.

**Fig. 2:**
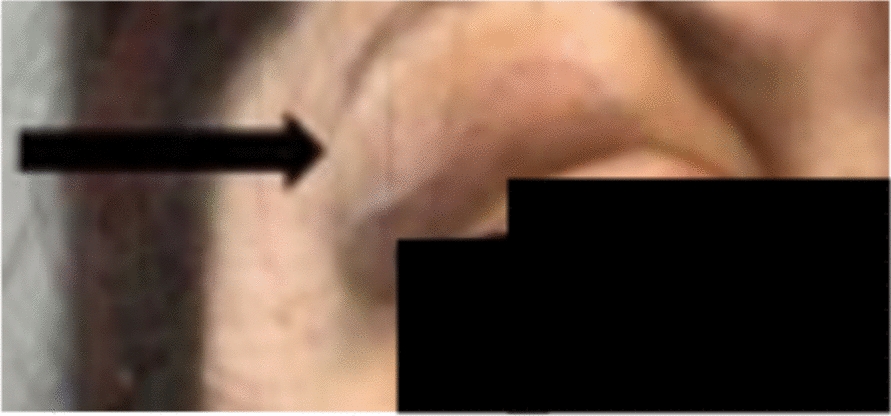
Swelling of the lacrimal gland

Initially, the patient declined both RDD-specific therapy and surgical intervention, and she did not attend follow-up appointments. Incompliance, particularly the failure to attend scheduled appointments, has been a recurrent issue throughout the course of the disease. She returned to our hemato-oncology department later in the end of 2022 with progressive periorbital and cervical swelling (Fig. 2). Complete blood count showed a mild microcytic, hypochromic iron deficiency anemia. Other routine laboratory analyses including liver and renal function tests as well as electrolytes were within normal ranges. Given the malignant imaging features of the parauterine mass, surgical management was prioritized by our gynecology team.

**Fig. 3:**
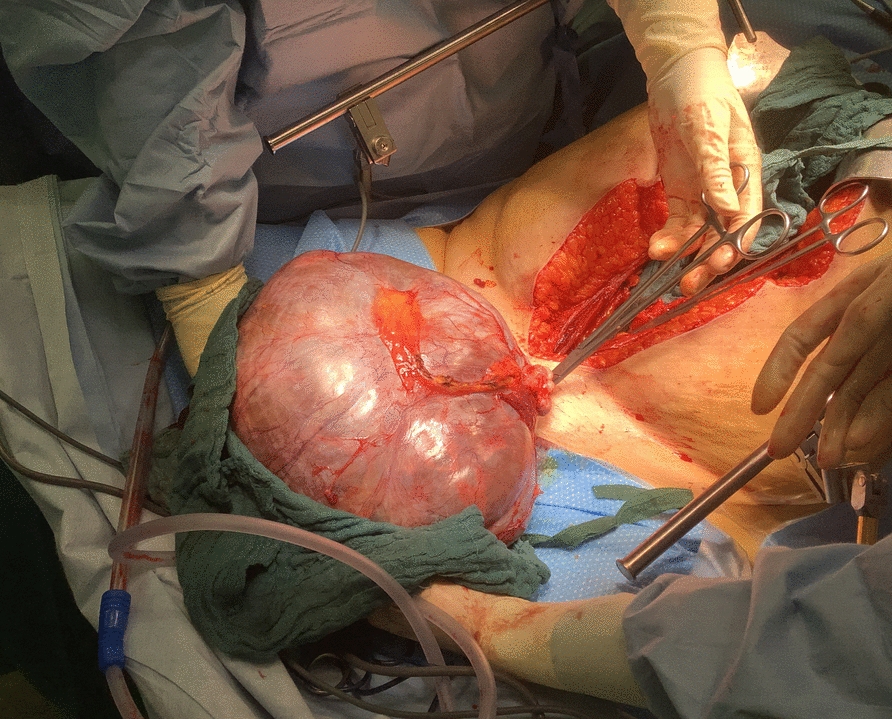
Tumor of the left ovary which was detected by positron emission tomography–computed tomography and had shown elevated glucose utilization at the margins. Pathological diagnosis: borderline tumor of the ovary with transition into endometrioid ovarian cancer

A median laparotomy with left adnexectomy and excision of a suspicious left para-iliac lymph node was performed in the beginning of 2023 after multidisciplinary discussion and airway assessment by ENT to ensure safe intubation. Intraoperatively, the tumor measured 25 cm and appeared to originate from the left ovary (Fig. 3). Frozen section analysis revealed a borderline ovarian tumor, while the lymph node biopsy showed RDD. The procedure was therefore concluded after the adnexectomy.

Final pathology reclassified the ovarian lesion as an endometrioid borderline tumor with transition into moderately differentiated endometrioid ovarian carcinoma. A second laparotomy was performed for hysterectomy, right adnexectomy, omentectomy, and para-iliac/para-aortic lymph node sampling. Radical lymphadenectomy was avoided owing to the lack of evidence on its safety in RDD. Final staging was pT1a, pNX, L0, V0, G2, R0. Molecular pathology of the RDD tissue revealed a* KRAS* mutation. Given the early stage of ovarian carcinoma, no adjuvant chemotherapy was initiated, and the patient was enrolled in tumor-specific follow-up.

For RDD, high-dose prednisolone (80 mg daily) was initiated preoperatively to bridge the time until relaparotomy in April of 2023, resulting in temporary reduction of eyelid swelling. After 4 weeks, symptoms worsened, and a 4-day dexamethasone pulse (4 mg daily) was administered. Owing to the *KRAS* mutation, MEK inhibition with cobimetinib was considered and a corresponding request for cost coverage was submitted to the patient’s health insurance provider, which was approved. Following the dexamethasone pulse, prednisolone was reintroduced and tapered. Owing to hyperglycemia and emerging dysphagia suggesting disease progression, therapy was switched to methotrexate (20 mg subcutaneously weekly) with folic acid rescue (5 mg the following day) and prednisolone (25 mg daily).

The patient then discontinued all treatment for several weeks, leading to further clinical progression, including worsening dysphagia and orbital and facial swelling. After complete recovery from the second laparotomy, she agreed to initiate cobimetinib at 60 mg daily in July 2023 (days 1–21 of a 28-day cycle) in accordance with international consensus recommendations. Severe acneiform dermatitis prompted a short discontinuation (2 weeks) of the medication and then a dose reduction to 20 mg for six cycles, followed by an alternating schedule of 40 mg and 20 mg daily since February 2024. The patient remains on this schedule of cobimetinib up to this date with a sustained therapeutic response, evaluated by clinical examination and magnet resonance imaging (MRI). The patient presents regularly (approximately every 6–8 weeks) to the hemato-oncological outpatient clinic for clinical examination and blood sampling. Thus far, she has shown very good tolerability to cobimetinib without any adverse effects. Laboratory tests occasionally revealed very mild lymphocytosis (up to 4.27/nL; reference range 1.22–3.56/nL), as well as mild elevations of lactate dehydrogenase (LDH, 260 U/L; reference range < 247 U/L) and creatine kinase (CK, 503 U/L; reference range < 170 U/L), currently without clinical significance. Whole-body MRI is performed approximately every 6 months, alternating with PET–CT scans twice yearly. The most recent imaging studies demonstrated a good therapeutic response with regressive-to-stable findings in the previously described manifestations. An overview of the patient’s clinical course is presented in Table 1.

**Table 1:** Overview of the patient’s clinical course over time regarding presentation, investigation, and treatment

Timeline	Presentation	Investigation	Treatment
2017 (ENT department, hemato-oncological department)	Restricted nasal breathing	Nasal polyposis diagnosed by sinonasal endoscopy	Pansinus surgery, pathology confirmed RDD, watch-and-wait strategy
2017–2022 (other hospitals)	Restricted nasal breathing, orbital, and cervical swelling, latent hyperthyroidism	Bone scintigraphy: tracer uptake in facial bones and distal femoral shafts Thyroid scintigraphy: hypofunctional areas without features of autoimmune thyroiditis	Pansinus surgery, partial mastoidectomy, conchotomy, cervical lymph node excision, partial cheek resection, maxillary sinus → histology: plasma cell- and histiocyte-rich infiltration
February and March 2022 (endocrinology department, hemato-oncology department, OBGYN department)	Referral because of latent hyperthyroidism, goiter Orbital and cervical swelling No palpable lymphadenopathy	Repeat thyroid scintigraphy: mild disseminated autonomy PET–CT: hypermetabolic RDD manifestations: intraorbital, right maxillary sinus, inferior nasal concha, right neck, thyroid extending into the mediastinum, medullary space of the lower extremities + hypermetabolic left parauterine mass (16.7 × 7.8 cm) and leftsided parailiac hypermetabolic mass	Denial of surgical intervention and/or RDD-specific therapy
October 2022	Progressive cervical and orbital swelling	Airway assessment via ENT	Prioritizing of gynecological surgical management
January 2023			Leftsided adnexectomy and parailiac lymphnode excision via longitudinal laparotomy
January 2023–March 2023	Temporary reduction of orbital swelling after prednisolone therapy, then progressive orbital swelling and dysphagia	Frozen section: parauterine mass classified as a borderline tumor, lymphnode classified as RDD, reclassification as moderately differentiated endometrioid ovarian carcinoma	4 weeks of high dose prednisolone (80 mg daily), 4 days of dexamethasone (4 mg) pulse therapy, then reintroduction of prednisolone and tapering to 25 mg daily, addition of methotrexate 20 mg weekly in March 2023
April 2023			Relaparotomy for surgical completion regarding the endometrioid ovarian cancer: hysterectomy, right adnexectomy, omentectomy, and para-iliac/para-aortic lymph node sampling
May 2023–June 2023	Progressive cervical and orbital swelling, progressive dysphagia		Discontinuation of all therapy through the patient
July 2023			Start of cobimetinib treatment 60 mg daily, 28 day cycle
August 2023	Development of acneiform dermatitis, clinical regression of cervical and orbital swelling, no dysphagia	Whole body MRI approx. every 6 months, alternating PET scans twice yearly, regular laboratory testing	Discontinuation of cobimetinib for 2 weeks, and the reintroduction with 20 mg of cobimetinib
August 2023—01/2024	Sustained remission		20 mg of cobimetinib
February 2024–today			Alternating 40 mg and 20 mg of cobimetinib, currently 27th cycle

## Discussion and conclusion

When the patient came to our hemato-oncological department in early 2022, the main problem for her was the extensive swelling of the eyes due to RDD of the lacrimal glands, which led to massive discomfort of the eye. Approximately 11% of RDD cases have orbital manifestation [6]. Head and neck involvement, also including the nasal cavity similar to our patient, has been reported in up to 22% of the patients [13]. Involvement of the bones is rare and only found in 5–10% of the cases, while manifestation in lymph nodes, similar to our patient, are common (57% of cases) [6].

The presence of multifocal disease, as found in our patient, is associated with mutations in the *KRAS* gene of the mitogen-activated protein kinase (MAPK) pathway, which was detected in our patient [14]. While the exact etiopathogenesis of RDD remains subject of research, the fact that mutations in the *KRAS* and *MAP2K1* gene are mutually exclusive, demonstrating the clonality seen in 33% of the patients with RDD [14], makes the previous perception that RDD is a reactive and non-neoplastic disorder questionable [15]. The detection of these mutations in at least a significant number of cases, might indicate that RDD is, in these cases, a neoplastic process [7]. In the conducted next generation sequencing panel from a biopsy of the lacrimal gland of our patient, not only a *KRAS* mutation was detected, but also low-level copy number variations (CNVs) of the gene DNA polymerase epsilon, catalytic subunit (POLE). Up to this date, there is no sufficient data concerning a causal association between CNVs in the gene *POLE* and RDD, but we found one case report of Proskuriakova *et al.* describing the case of a man with RDD where genetic testing also revealed mutations in the genes *POLE* and *KRAS* [16].

There is only limited data about the association of RDD and cancer, especially gynecological cancer, similar to our case with ovarian cancer. However, an association with neoplasia is known [5]. Concerning gynecological cancers, there are case reports of simultaneous cutaneous RDD and cervical cancer [17] and the involvement of RDD of the chest wall after mastectomy as treatment for breast cancer [18]. RDD can also involve the female genital organs, such as the uterus and the ovaries without concomitant other neoplasias [19, 20]. Concerning nongynecological cancers, RDD has been associated with Hodgin’s and non-Hodgkin’s lymphoma [21], clear cell sarcoma [22], and has also been reported after bone marrow transplantation and myelodysplastic syndrome [23, 24]. Whether or not there is a causal link between RDD and malignancies remains a subject of research. Regarding the simultaneous endometrioid ovarian cancer in our patient, there have been no previous reports on a possible link between ovarian cancer and RDD to our best knowledge. Endometrioid ovarian cancer is often associated with endometriosis and tends to present in an early stage compared with serous cell ovarian cancer [25]. Our patient had no previous diagnosis of endometriosis. While somatic *KRAS* mutations are more often seen in patients with mucinous ovarian cancer [26], endometrioid ovarian cancer is linked to mutations in the AT-rich interactive domain-containing protein 1A (*ARID1A*) gene, a tumor suppressor gene [27, 28].

Moreover, our patient did not have any pathological findings in the autoimmune disease panel, which was carried out because of possible simultaneous autoimmune diseases in patients with RDD [29].

Since a mutation in the *KRAS* gene of the MAPK pathway was detected in our patient, and because of the extensive extranodal disease in various locations and relapse during treatment with corticosteroids and methotrexate, a therapy with the MEK inhibitor cobimetinib was chosen [5]. MEK inhibition has shown good preliminary results in patients with *KRAS* mutation, leading to remission and complete response [11, 30]. A phase 2 trial of the use cobimetinib in histiocytotic disorders was completed in December 2022 (www.clinicaltrials.gov, Identifier: NCT02649972), which led to US Food and Drug Administration (FDA) approval [31]. Previously, a proof of concept study carried out by Diamond *et al.* showed a response rate of 100% in 18 treated patients with histiocytotic disorders and mutations in the MAPK pathway after 1 year [32].

While it is proven that extranodal involvement in the rare Rosai Dorfman disease is not uncommon, a possible causal link between RDD and cancer remains a subject of research [5]. There are case reports of simultaneous cancer and RDD [17, 21–23]. RDD itself is often described as a reactive process, but since it can be associated with mutations involving the MAPK signaling pathway it could—in these cases—also be described as a neoplastic process [7]. In our patient, after treating the simultaneous endometrioid ovarian cancer, a treatment with the MEK inhibitor cobimetinib seemed to be the best option for this patient, because of a detected *KRAS* mutation and extensive disease with involvement of various locations (head and neck region, parailiacal lymph node, lacrimal gland, bone shafts). Further studies with a larger sample size regarding the use and efficacy of cobimetinib in patients with RDD and regarding a possible causal association between cancer and RDD are needed to draw further conclusions.

## Supplementary Information

Additional file 1.

## Data Availability

All cited literature and data was found on https://pubmed.ncbi.nlm.nih.gov/.

## Abbreviations

RDD: Rosai Dorfman disease
MEK: Mitogen-activated protein kinase kinase
KRAS: Kirsten rat sarcoma virus
ENT: Ear nose and throat
Mg: Milligrams
PET–CT: Positron emission tomography–computed tomography
MRI: Magnetic resonance imaging
MAPK: Mitogen-activated protein kinase
CNVs: Copy number variations
POLE: DNA polymerase epsilon, catalytic subunit
ARID1A: AT-rich interactive domain-containing protein 1A
FDA: US Food and Drug Administration
