# Supplementary material for: Multifocal Rosai Dorfman disease and simultaneous endometrioid ovarian cancer: a case report
Source: J Med Case Rep. 2026 Mar 31;20:179. doi: 10.1186/s13256-025-05772-4 (PMC13067726; doi:10.1186/s13256-025-05772-4)
Supplement: Supplementary file 1 — Additional file 1. [file 13256_2025_5772_MOESM1_ESM.pdf]

CARE case report guidelines

CARE Checklist of information to include when writing a case report

(cc) BY-NC-ND

✓

Topic	Item	Checklist item description	Reported on Line
Title	1	The diagnosis or intervention of primary focus followed by the words "case report"	1 - 2
Key Words	2	2 to 5 key words that identify diagnoses or interventions in this case report, including "case report"	53
Abstract (no references)	3a	Introduction: What is unique about this case and what does it add to the scientific literature?	36 – 43
	3b	Main symptoms and/or important clinical findings	44 – 48
	3c	The main diagnoses, therapeutic interventions, and outcomes	
	3d	Conclusion—What is the main "take-away" lesson(s) from this case?	49 – 52
Introduction	4	One or two paragraphs summarizing why this case is unique (**may include references**)	55 – 75
Patient Information	5a	De-identified patient specific information.	78
	5b	Primary concerns and symptoms of the patient.	107, 79, 130
	5c	Medical, family, and psycho-social history including relevant genetic information	78 - 91, 150 - 155
	5d	Relevant past interventions with outcomes	78 – 95
Clinical Findings	6	Describe significant physical examination (PE) and important clinical findings.	92 – 104
Timeline	7	Historical and current information from this episode of care organized as a timeline	78 – 135
Diagnostic Assessment	8a	Diagnostic testing (such as PE, laboratory testing, imaging, surveys).	79, 89-90, 92-93, 98-99, 104
	8b	Diagnostic challenges (such as access to testing, financial, or cultural)	105-106, 109 – 111
	8c	Diagnosis (including other diagnoses considered)	99, 115 – 116
	8d	Prognosis (such as staging in oncology) where applicable	119, 135, 121, 179, 180
Therapeutic Intervention	9a	Types of therapeutic intervention (such as pharmacologic, surgical, preventive, self-care)	105, 109 – 114, 112-129, 130-134
	9b	Administration of therapeutic intervention (such as dosage, strength, duration)	122, 124, 128-129, 131-132
	9c	Changes in therapeutic intervention (with rationale)	116-119, 123-127, 131-133
Follow-up and Outcomes	10a	Clinician and patient-assessed outcomes (if available)	135
	10b	Important follow-up diagnostic and other test results	119-121, 99, 135
	10c	Intervention adherence and tolerability (How was this assessed?)	130-132
	10d	Adverse and unanticipated events	131-133
Discussion	11a	A scientific discussion of the strengths AND limitations associated with this case report	136 – 197
	11b	Discussion of the relevant medical literature **with references**.	136 – 197
	11c	The scientific rationale for any conclusions (including assessment of possible causes)	136 – 197
	11d	The primary "take-away" lessons of this case report (without references) in a one paragraph conclusion	191 – 197
Patient Perspective	12	The patient should share their perspective in one to two paragraphs on the treatment(s) they received	∅ The patient did not wish to share their perspecti[illegible]
Informed Consent	13	Did the patient give informed consent? Please provide if requested	Yes ☒ No ☐
